# Identification of early biomarkers in saliva in genetically engineered mouse model C(3)1-TAg of breast cancer

**DOI:** 10.1038/s41598-022-14514-1

**Published:** 2022-07-07

**Authors:** Isadora Fernandes Gilson Sena, Larissa Lessi Fernandes, Leonardo Lima Lorandi, Thais Viggiani Santana, Luciana Cintra, Ismael Feitosa Lima, Leo Kei Iwai, Jill M. Kramer, Alexander Birbrair, Débora Heller

**Affiliations:** 1 Department of Pathology, Federal University of Minas Gerais, Belo Horizonte, Minas Gerais Brazil; 2 Post Graduate Program in Dentistry, Cruzeiro do Sul University, São Paulo, Brazil; 3 Hospital Israelita Albert Einstein, São Paulo, Brazil; 4 Institute of Biomedical Sciences, University of São Paulo, São Paulo, Brazil; 5 Laboratory of Applied Toxicology, Center of Toxins, Immune-Response and Cell Signaling (LETA/CeTICS), Instituto Butantan, São Paulo, Brazil; 6 Department of Oral Biology, School of Dental Medicine, The University of Buffalo, State University of New York, Buffalo, NY USA; 7 Department of Dermatology, Medical Sciences Center, University of Wisconsin-Madison, Rm 4385, 1300 University Avenue, Madison, WI 53706 USA; 8 Department of Radiology, Columbia University Medical Center, New York, NY USA; 9 Department of Periodontology, University of Texas Health Science Center San Antonio, San Antonio, TX USA

**Keywords:** Cancer, Molecular biology, Oncology, Biomarkers, Diagnostic markers

## Abstract

Breast cancer is one of leading causes of death worldwide in the female population. Deaths from breast cancer could be reduced significantly through earlier and more efficient detection of the disease. Saliva, an oral fluid that contains an abundance of protein biomarkers, has been recognized as a promising diagnostic biofluid that is easy to isolate through non-invasive techniques. Assays on saliva can be performed rapidly and are cost-effective. Therefore, our work aimed to identify salivary biomarkers present in the initial stages of breast cancer, where cell alterations are not yet detectable by histopathological analysis. Using state-of-the-art techniques, we employed a transgenic mouse model of mammary cancer to identify molecular changes in precancerous stage breast cancer through protein analysis in saliva. Through corroborative molecular approaches, we established that proteins related to metabolic changes, inflammatory process and cell matrix degradation are detected in saliva at the onset of tumor development. Our work demonstrated that salivary protein profiles can be used to identify cellular changes associated with precancerous stage breast cancer through non-invasive means even prior to biopsy-evident disease.

## Introduction

Breast cancer is the most common cancer in the world population and the leading cause of cancer-related death in woman^1^. Breast cancer-related morbidity and mortality could be diminished if the population had access to early diagnosis and effective treatments. The early detection of breast cancer is a crucial factor in improving patient survival rate^2^. Conventional screening (physical examination and mammography) has a lower-than-desirable sensitivity and specificity, yet screening mammography is considered the gold standard for detecting breast cancer. Indeed, it is estimated that screening mammography detects pathology in between 54 and 77% of cases, depending on the type of mammographic procedure and this exam can generate unnecessary biopsies, increase the cost of public and private health services, in addition to exposing women unnecessarily to radiation^3–5^. Thus, it is imperative for the scientific community to develop alternative diagnostic methods that allow the early detection of breast cancer in a more efficient and easily accessible way^6^.

Saliva, an oral fluid that contains an abundance of protein biomarkers and genetic molecules, has been recognized as a promising biological material for early detection of disease^6,7^. Because it is easy and inexpensive to sample with minimal discomfort, oral fluid is an excellent source of potential biomarkers, and this has important public health relevance^2^. A wide range of salivary biomarkers are reported. Of particular significance to this study, saliva has been used to detect breast cancer in patients with an established diagnosis with a sensitivity and specificity ranging from 50 to 97%^8–12^. CA-15-3 is a transmembrane glycoprotein present in the sera that is used to detect advanced breast cancer^13^, and levels of this biomarker have been shown to be significantly higher in cancer patients^8,11^. Of note, a positive correlation between CA-15-3 levels in serum and saliva is observed^14^. In addition, elevated levels of c-erbB-2, EGFR, Cathepsin-D and p53 were observed in the saliva and serum of patients with breast cancer. Likewise, growth factors such as epidermal growth factor (EGF) and vascular endothelial growth factor (VEGF) were more abundant in the saliva of breast cancer patients^9,15^.

However, most salivary biomarkers identified detect advanced stages of breast cancer more accurately than early stages. Thus, there is a lack of evidence for the use of salivary biomarkers in the early diagnosis of breast cancer, and further research is needed to elucidate potential new biomarkers^6^. This study utilized a well-established murine model of endogenous breast tumors to enable discovery of biomarkers in the earliest stages of cancer^16^. A major advantage of this murine model is the ability to analyze biological fluids at defined periods at very early stages of tumor development, even before the appearance of an overt tumor mass^17^.

The C3(1)/SV40/T-Antigen (C3(1)-TAg) mouse model is a genetically-engineered mouse model (GEMM) that exhibits spontaneous mammary tumor development within the breast microenvironment^16^. This is an excellent model for breast cancer, because it recapitulates the human disease in a number of ways and allows for analysis of tumors at early disease stages^18^. Furthermore, in a seminal consensus report from a meeting convened by the United States National Institutes of Health (NIH), pathologists and veterinarians highlighted the similarity between tumors that arise in the C3(1)-TAg model and human pathology, including the presence of ductal carcinoma in situ (DCIS) type with sclerosing stroma^19^. Therefore, we used this mouse model to identify salivary biomarkers in mice with precancerous stage breast cancer, even prior to histopathological detection of disease. Our results show that the identification of proteins through saliva, a non-invasive and easily collected biofluid, may be a promising technique for the detection of biomarkers in precancerous stages of breast cancer.

## Results

### C(3)1-TAg animals at 4-weeks old have a similar histology to wild-type animals and does not show any cellular alterations in histology

**Figure 1:**
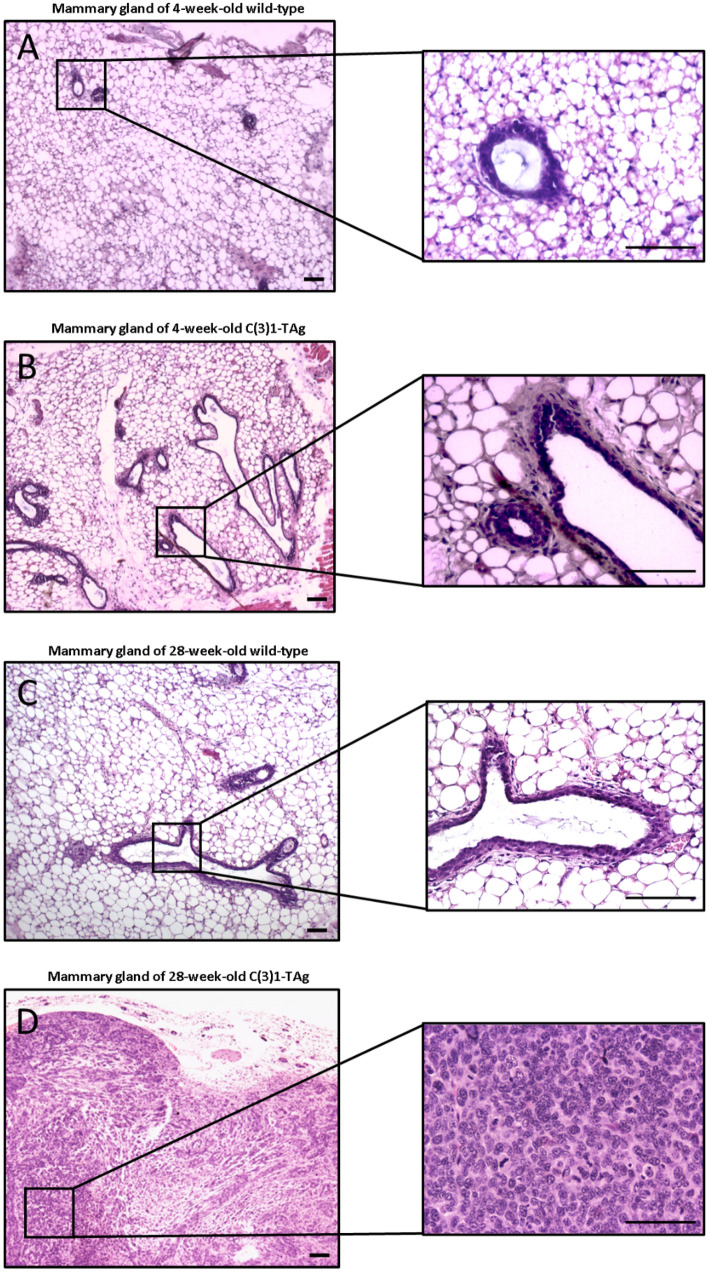
Characterization of carcinoma development in C(3)1-TAg and wild-type mice. (**A**) Mammary tissue from 4-week-old wild-type and (**B**) 4-week-old C(3)1-TAg mice (**C**) Mammary tissue from 28-week-old wild-type and (**D**) 28-week-old C(3)1-TAg. Low and high power magnifications are shown. Tissue from one representative animal of each group is shown. Scale bar: 50 μm.

We initially began our study by identifying the histopathological disease progression in mammary tissue in C3(1)-TAg females. We euthanized females at 4 and 28 weeks of age (n = 3 each), harvested mammary tissues, and examined formalin-fixed H&E stained tissue sections. We performed parallel analyses in C57BL/6J age and sex-matched controls (n = 3 each). In accordinance with our previous findings^18^, mammary tissue from 4-week-old C3(1)-TAg animals exhibited normal cellular architecture. Numerous mammary ducts with one or two layers of epithelial cells surrounded by myoepithelial cells in a rich adipose tissue were observed, and tissues were devoid of any pre-malignant or malignant changes (Fig. 1A,B). Nevertheless, at the age of 28 weeks, however, mammary tissue from C3(1)-TAg mice displayed invasive carcinoma, with a robust proliferation of hyperchromatic cells exhibiting mitotic figures, condensed chromatin and prominent nucleoli in C3(1)-TAg females (Fig. 1D) compared to the mammary tissue of the wild-type animal that did not show any pre-malignant or malignant changes (Fig. 1C). Thus, we established that C3(1)-TAg mice at 4 weeks display normal mammary tissue, whereas advanced disease is evident at 28 weeks.

### Qualitative proteomic analysis of saliva derived from 4-week-old C3(1)-TAg mice versus 4-week-old wild-type mice

**Figure 2:**
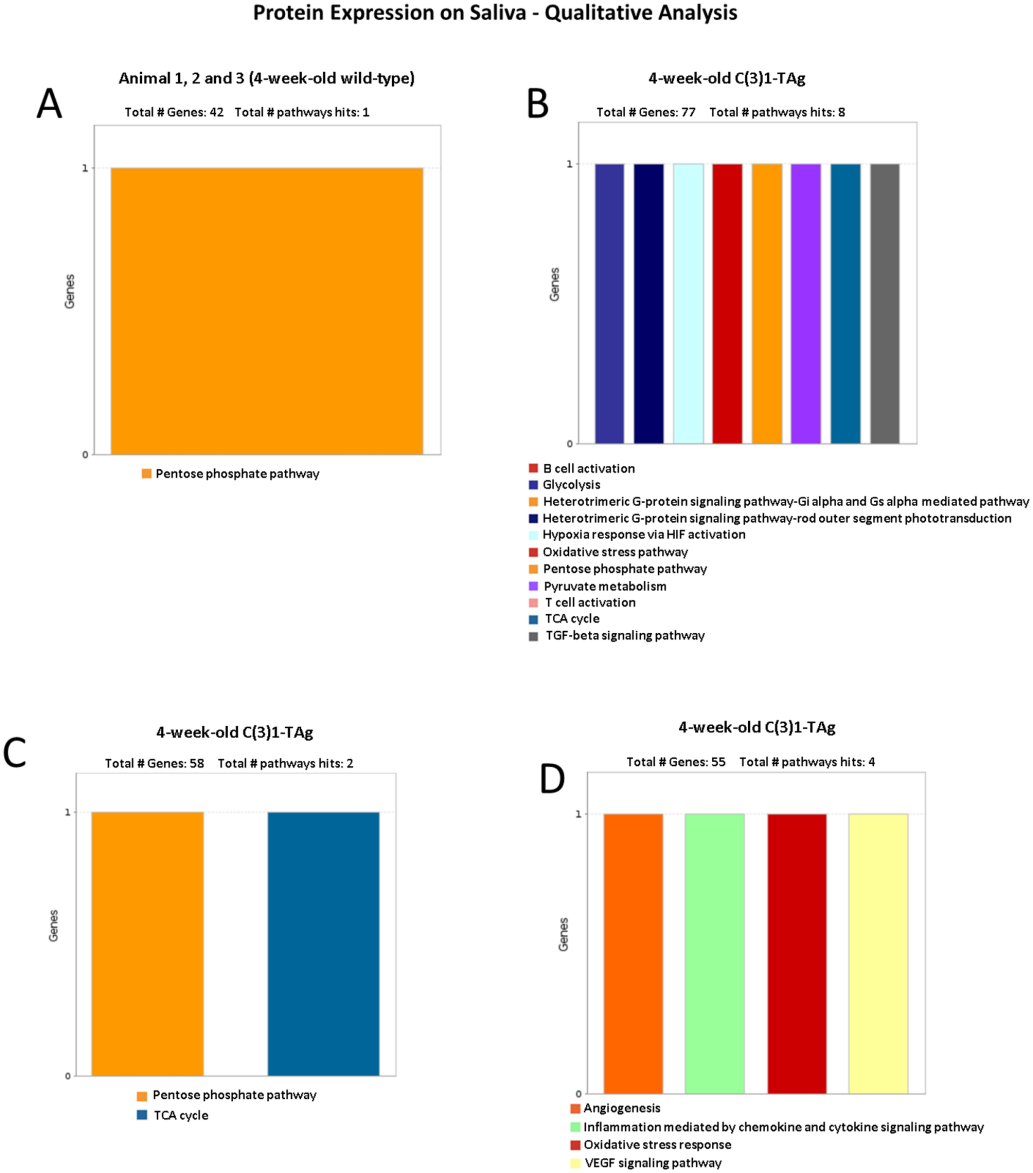
Qualitative analysis of the main proteins pathways expressed in the saliva samples of 4-week-old wild-type mice (**A**) versus (**B**–**D**) C3(1)-TAg mice. All three wild-type animals had the same protein pathway expression and are represented by Fig. 1A. Four-week-old C(3)1-TAg animals 1, 2 and 3 presented complex pathways and are represented separately (Fig. 1B–D).

To search for salivary biomarkers present prior to the detection in histological analysis of breast cancer, we collected the saliva of 4-week-old C3(1)-TAg (n = 3) and 4-week-old wild-type mice (n = 3). A mean total of 139 ± 24 and 124 ± 39 proteins were identified in saliva of 4-week-old wild-type mice and 4-week-old C3(1)-TAg mice, respectively. We first performed a qualitative proteomic analysis of the samples using Panther software. Interestingly, saliva from all three 4-week-old wild-type mice presented the same protein pathway expression with a highly enriched for expression of proteins in the pentose phosphate pathway (Fig. 2A). These proteins are normally expressed in adipose and mammary tissue due to the high fatty acid synthesis^20^. In contrast, analysis of saliva from 4-week-old C3(1)-TAg females revealed expression of proteins related to angiogenesis, inflammatory process and oxidative stress (Fig. 2B–D).

### Gastric triacylglycerol lipase (GTL) and submandibular gland protein C (SMGC) are elevated in saliva derived from 4-week-old C3(1)-TAg mice compared to age and sex-matched controls

**Figure 3:**
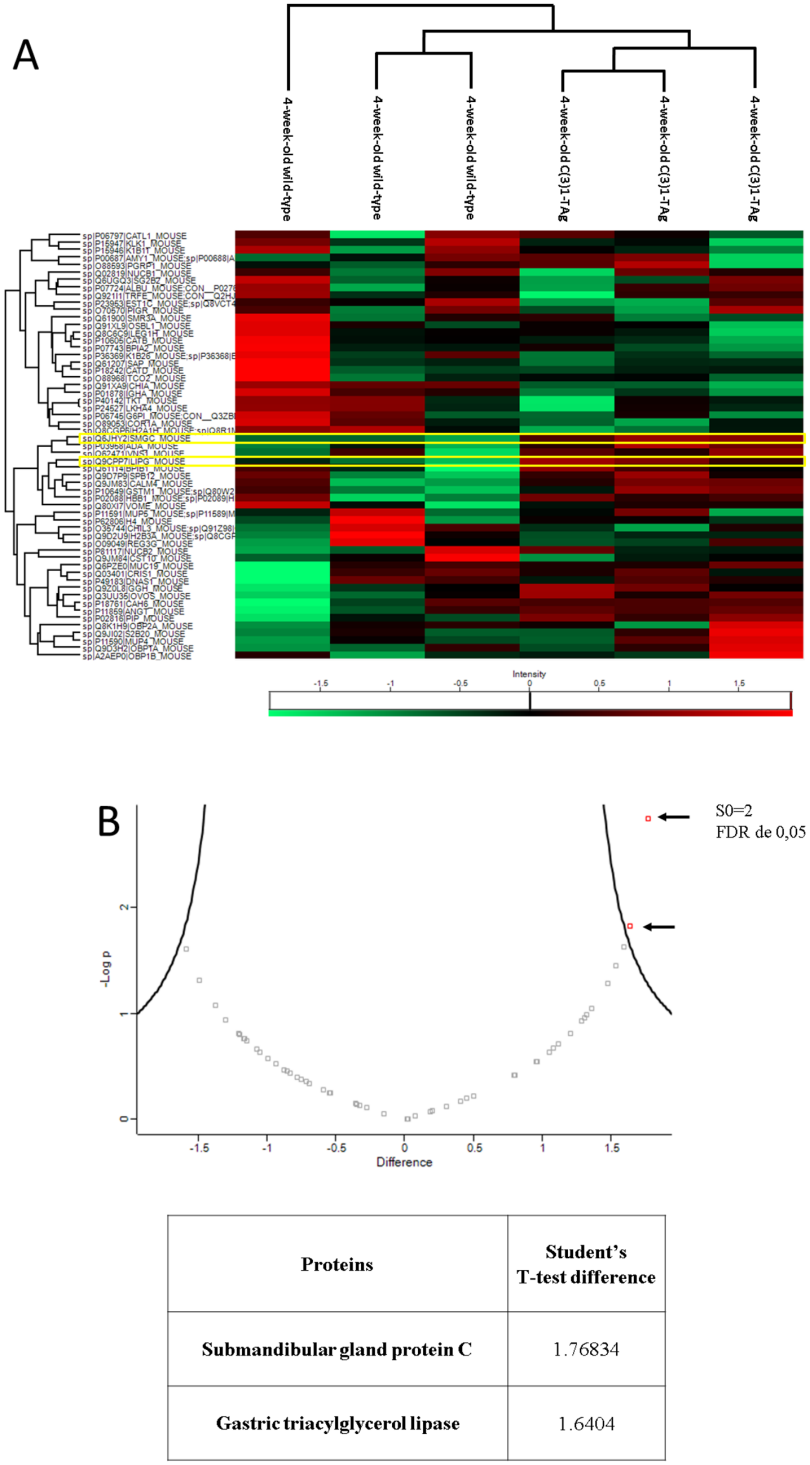
Protein expression in saliva of 4-week-old C(3)1-TAg females compared to age-matched wild-type. (**A**) Heatmap of protein expression in saliva comparing 4-week-old C(3)1-TAg with 4-week-old wild-type animals. The two proteins that are significantly higher expressed on 4-week-old C(3)1-TAg animals compared to 4-week-old wild-type mice are highlight in yellow. (**B**) Volcano plot showing two proteins (represented by red dots with arrows. Each red dot represents a separate gene) that are significantly higher expressed on 4-week-old C(3)1-TAg animals compared to 4-week-old wild-type mice.

Using quantitative proteomic analysis, we identified two salivary proteins that were significantly increased in C(3)1-Tag mice as compared to wild-type controls at the 4-week time point, gastric triacylglycerol lipase (GTL) and submandibular gland protein C (SMGC) (Fig. 3). GTL is a protein expressed by the LIPF gene and contributes to the metabolism of adipose tissue, favoring a catabolic state that assists the proliferation of tumor cells^21^. This finding corroborates with the qualitative analysis that demonstrated an expression of proteins related to lipid metabolism, such as tricarboxylic acid (TCA) cycle (Fig. 2B,C). Submandibular gland protein C is usually expressed only in neonatal and young mice and it is expressed more highly in female mice^22^. Interestingly, SMGC is related to Mucin-19, a protein that has a higher expression in breast cancer cell, and its expression is correlated with a worse prognosis in human^23,24^ and that we found it was also highly express in C(3)1-TAg animals with invasive carcinoma (Fig. 5). Also, LIPF and MUC19, the gene that expresses SMGC, was associated with a worse survival probability in patients with breast cancer (Fig. S1). Thus, our results identified two that are putative biomarkers for precancerous stage breast cancer.

### Animals with invasive carcinoma express proteins related to oxidative stress and inflammation

**Figure 4:**
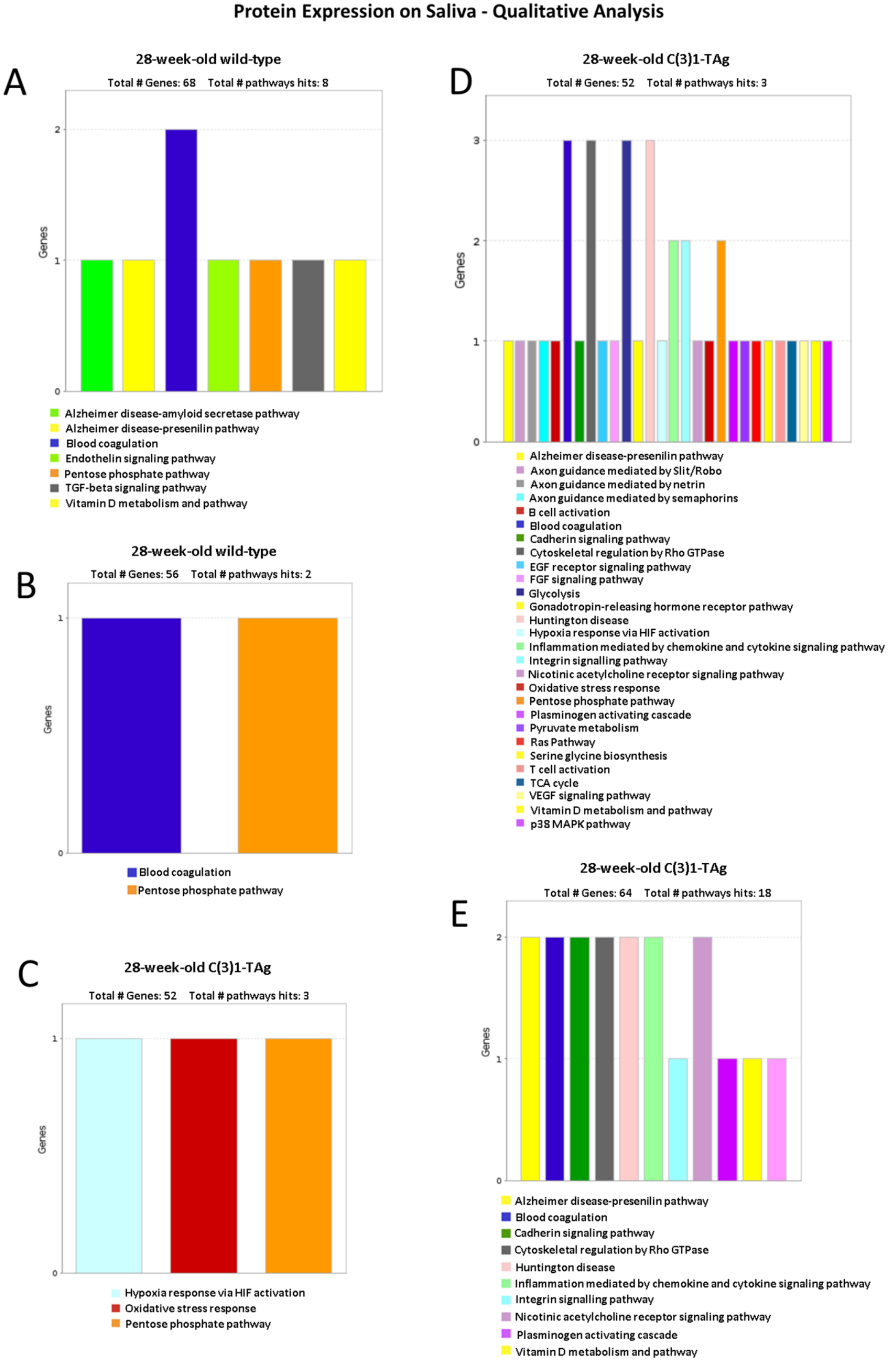
Qualitative pathway analysis of proteins expressed in the saliva of 28-week-old wild-type mice (**A**,**B**) and (**C**–**E**) C3(1)-TAg mice. The graphs obtained by the Panther software show a singular profile of protein expression of each animal, with one wild-type mice that could not be classified in Panther software.

To compare early stage disease findings to those with animals with invasive carcinoma, a late stage of breast cancer, we collected the saliva in 28-week-old C3(1)-TAg and 28-week-old wild-type mice. A mean total of 198 ± 74 and 134 ± 7 proteins were identified in saliva of 28-week-old C3(1)-Tag mice and 28-week-old wild-type mice, respectively. First, we performed qualitative analysis to identify the main proteins pathways expressed in the saliva samples using Panther software. Saliva from 28-week-old wild-type mice was enriched for proteins related to pentose phosphate and blood coagulation (Fig. 4A,B). In contrast saliva from 28-week-old C3(1)-TAg animals showed elevated protein expression pathways related to oxidative stress and inflammation (Fig. 4C–E). Thus, our results demonstrated that analysis of proteins in saliva may indicate cellular processes related to breast cancer.

### Animals with precancerous stage disease express more proteins related to cell matrix degradation while animals with invasive carcinoma express proteins related to the immune system

**Figure 5:**
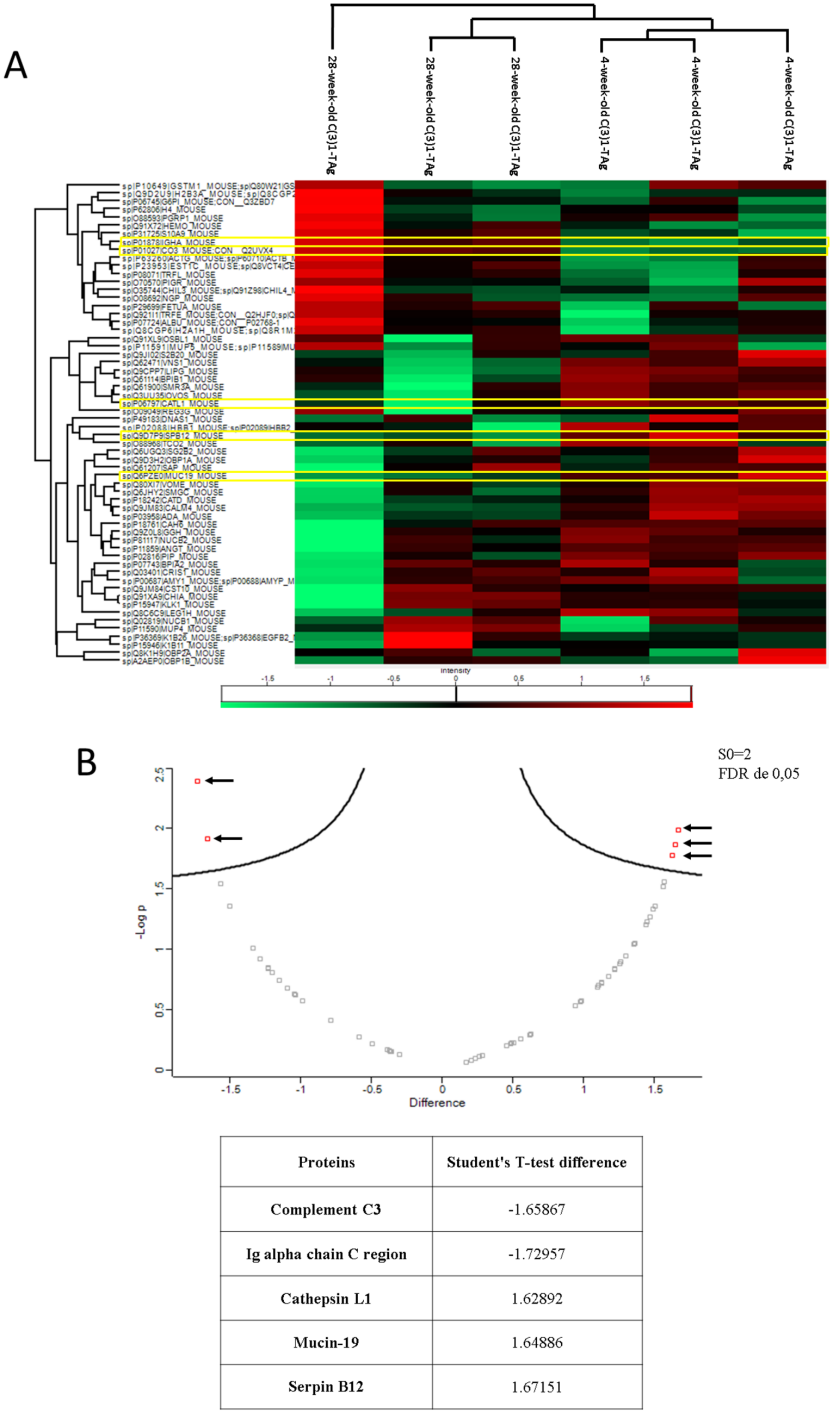
Differential protein expression in the saliva of C3(1)-TAg animals at 4-week and 28-week time points. (**A**) Heatmap comparing 4-week-old and 28-week-old C(3)1-TAg. The proteins that differ significantly between 4-week-old C(3)1-TAg animals and 4-week-old wild-type mice are highlight in yellow. (**B**) Volcano plot showing five proteins (represented by red dots with arrows. Each red dot represents a separate gene) that are expressed differently between animals with mammary cancer in the initial stage and animals with the invasive carcinoma. A negative result demonstrates that the protein is more expressed in the 28-week-old C(3)1-TAg samples and a positive result demonstrates that the protein is more expressed in the 4-weeks-old C(3)1-TAg.

Analyses using Panther software showed that 28-week-old C3(1)-TAg mice have a greater complexity of expression of salivary proteins (Fig. 4C–E) when compared to 4-week-old C3(1)-TAg mice (Fig. 2B-D). Thus, in order to verify if the proteins in saliva in the initial stage were altered during the course of tumor progression, we compared the saliva samples of young and aged C3(1)-TAg animals (Fig. 5). We found that cathepsin L1, serpin B12 and mucin-19 were highly expressed in mice at the initial stage of disease, whereas Ig Alpha Chain C Region (IGHA) and Complement C3 were enriched in animals with invasive carcinoma. Therefore, our studies indicate that at the beginning of tumor development proteins related to cell matrix degradation are upregulated, and in a more advanced stage of cancer proteins related to immunity are elevated in saliva.

## Discussion

Using a transgenic animal model that allows the study of mammary cancer in early stage disease, we were able to identify differential protein expression in saliva of 4-week old C3(1)-Tag when compared to 4-week old wild-type mice, even though the breast histology was similar between the two groups.

The C3(1)-TAg females on the C57BL/6 J background are triple-negative due the low expression or lack of ER, PR, and HER2 within these tumors which is commonly associated with more aggressive tumors^20^. We have previously described^18^ the breast cancer progression in this mouse model. Briefly, at 4 weeks and 8 weeks of age, there are no cellular alterations in histology. At 12 weeks of age, there is cells hyperplasia. At 16 weeks and 20 weeks, mammary intraepithelial neoplasia is identified and invasive carcinoma starts at 24 weeks of age. We decided to analyze the time point of 4 weeks old since the animal already has the genetic alteration with the inhibition of p53 in the breast tissue, that favor the uncontrolled proliferation of the cells, but does not show cellular alteration on histology, compared to the 28 weeks old animal that already has advanced disease.

Of interest, at 4-weeks old C3(1)-TAg mice we were able to identify potential biomarkers that could already be related to tumor metabolism pathways. Proteins involved in the TCA cycle, pyruvate metabolism and glycolysis were more expressed in the saliva of C(3)1-TAg mice and were present prior to mammary tumor development. The TCA cycle is important for energy metabolism and some studies show that alteration in this cycle may be related to cancer^25,26^. For instance, Lu and colleagues demonstrated that the metabolic profile of tumor cells can be related to the aggressiveness of cancer^27^.

Previous studies have detected salivary biomarkers in patients with breast cancer, such as CA15-3 and CA-125^11,12^. However, these studies only analyzed samples from patients with advanced tumors. To the best of our knowledge, this is the first study to show detection of potential protein biomarkers at precancerous stages of tumor development, before any histological change is detected.

The diagnosis of cancer at an early stage positively impacts prognosis, treatment and survival rates^28^. In the present study, the quantitative analysis showed that 4-week old C(3)1-TAg animals had a higher expression of GTL when compared to control animals. Cells with high rate of proliferation require significant amounts of energy, such as ATP, nucleotides and lipids. Lipases aid in the entry of fatty acids into cells where they are used in metabolic pathways^29^. Furthermore, it is known that high levels of lipid catabolism can promote lipotoxic effects, including in skeletal muscle, which favors cachexia in cancer patients^21^. GTL is highly expressed in gastric cancer and related lipases are highly expressed in testicular and breast cancer^30–32^. Moreover, Pang-Kuo Lo and colleagues identified that women with basal-type triple-negative human breast cancer have a higher expression of endothelial lipase, a lipoprotein lipase belonging to the triglyceride lipase gene family, in comparison with other types of cancer^33^. Our study corroborates with these findings, indicating a higher expression of GTL in saliva in animals at precancerous stage (4-week old C(3)1-TAg compared to age-matched wild-type.

Also, the present study revealed differences in salivary protein expression between pre-disease mice and those with invasive carcinoma. Of interest, Cathepsin L1, Serpin B12, and Mucin-19 were upregulated prior to disease development. Cathepsin L1 and serpin B12 are proteases that are upregulated in many cancers and are correlated to tumor invasion^34,35^. Cathepsin L1 facilitates the degradation of extracellular matrix and this promotes tumor cell detachment and metastasis and it is known to be overexpressed in many cancers, such as pancreatic, gastric, breast and ovarian malignancies^34^. Furthermore, cathepsin L1 has been previously identified as one of the most highly expressed proteins in breast cancer tissue^36^. Serpin B12 was also significantly expressed in saliva in animals at initial stage of cancer. An increased expression of this protein has been observed in ovarian cancer, suggesting its identification as a potential biomarker for early detection of ovarian carcinomas^37^. In addition, IGHA and complement C3 were upregulated in saliva from mice with advanced disease. These proteins were previously identified as candidate biomarkers in glioblastoma, bladder and breast cancer, including in triple-negative breast cancer^38–40^. Hence, our data show the potential utility of salivary proteome analyses for early detection of breast cancer.

Due to the high incidence of breast cancer throughout the world, the use of in vivo models to identify, classify and characterize tumors are invaluable. Many different types of models are available, including those that use grafted tissues derived from mouse or human cell lines and genetically modified mouse models^41,42^. Each mouse model has advantages and disadvantages, and the selection of an appropriate model to investigate breast cancer is an important decision that will influence the interpretation of research results^18^. For example, a limitation in xenograft and allograft models is the rapid development of the tumor, due to the high aggressiveness of the tumor that are injected in the animal. Thus, it is difficult to study the evolution of the disease from its beginning. Furthermore, most studies inject the tumor cells into the flank or into another region that is not the cell's native environment, which can influence tumor development and response^43^.

In contrast, GEMMs have a slow tumor progression that allows for the study of entire evolution of the tumor, including the early stages of cancer. The tumor progression occurs in the natural microenvironment of the cancer cell and the mice have an immune system intact^49–86^. However, this model includes an extensive breeding program that requires a long time and cost^43^.

A limitation of our study was the small sample size, in addition to only analyzing one GEMM model. Therefore, future studies are needed with larger sample sizes to validate our findings. Additionally, evaluation of other GEMMs, such as the p53-null-T11^44^ and *Apc*^1572T/+^^45^ strains are needed to determine whether our findings extend to other models of breast cancer. Finally, this is a pre-clinical study, and further studies in large patient cohorts are necessary to validate and extend our findings.

In conclusion, our results show that the identification of proteins through saliva, a non-invasive and easily collected biofluid, can be a promising technique for the detection of potential biomarkers in early stages of breast cancer. This study lays the groundwork for future studies that aim to identify robust salivary biomarkers for breast cancer.

## Materials and methods

### Animals

C3(1)-TAg female mice on the C57BL/6J genetic background were obtained from the Tissue Microenvironment Laboratory of the Federal University of Minas Gerais^18^ and C57BL/6J wild-type mice were obtained from the Central Animal Housing of the Federal University of Minas Gerais. All mice were housed in a pathogen-free facility of the Animal Research Program at the Federal University of Minas Gerais under a controlled light cycle (12:12-h light/dark cycle) and fed ad libitum. All experiments were performed in accordance with relevant guidelines and regulations. All procedures were performed with the approval of the Animal Use Ethics Commission of the Federal University of Minas Gerais (CEUA/UFMG) (Protocol 204/2017). This study was carried out in compliance with the ARRIVE guidelines.

### Experimental design

In order to perform the analysis of protein expression on saliva and the histological analysis C3(1)-TAg females in the C57BL/6J background were used as the experimental group and age-matched C57BL/6J wild-type mice as controls. First, the saliva was collected in animals at 4-weeks old (n = 3), for not yet present any cellular alterations on histopathology, and 28-weeks (n = 3) for presenting invasive carcinoma^16,18^. After that, the same animals were euthanized and the mammary tissue collected.

### Genotyping of TAg

DNA extraction was performed using the phenol–chloroform method as described previously^46^. Proteinase K were used for the lysis phase, phenol–chloroform-isoamyl alcohol were used for the wash phase, DNA precipitation occurred with ice cold ethanol and DNA elution with nuclease-free water. Then, a conventional polymerase chain reaction (PCR) was performed to amplify the gene of interest, as previously described^18^, using the Applied Biosystems MiniAmp thermocycler (ThermoFisher Scientific Inc, Massachusetts, USA). The primers used were: Forward: 5 'CAGAGCAGAATTGTGGAGTGG-3' and Reverse: 5'-GGACAAACCACAACTAGAATGCAGTG -3'. The amplified product was 500 bp and identified by 1% agarose gel electrophoresis.

### Saliva collection

The animals were anesthetized with a mixture of 114 mg/kg ketamine and 17 mg/kg xylazine. After the animals were anesthetized, salivation was induced peritoneally by administering 10 mg/kg of pilocarpine (Sigma-Aldrich) in phosphate buffered saline. Saliva was collected with a pipette for a maximum period of 10 min and transferred to a 1.5 ml microtube. The samples were kept on ice during the collection procedure, and immediately after collection, the microtubes containing saliva were centrifuged at 14000xg for 15 min at 4 °C (Eppendorf, centrifuge 5427R). The supernatant resulting from this process was stored in a freezer -80 °C until mass spectrometry was performed.

### Collection of mammary tissue and histological examination

Mammary tissue was harvested from mice at 4 (n = 3) and 28-weeks of age (n = 3) following euthanasia^18^. Euthanasia was performed by cervical dislocation followed by total resection of mammary glands. The tissues were fixed in 10% (v/v) buffered formalin, embedded in paraffin blocks, sectioned into a 4 μm thickness, placed onto glass slides, and stained with hematoxylin–eosin. The whole tissue of each animal was analyzed at 20X magnification.

### Microscopic analysis

A BX51 microscope (Olympus, Media Cybernetics, United States) equipped with Image-Pro Express 4.0 software (Media Cybernetics, United States) with a resolution of 1,392 × 1,040 pixels was used to obtain images for histopathologic analysis.

### Kaplan–Meier curves

Survival curves were plotted using the website Kaplan Meier plotter (https://kmplot.com/analysis/) (Fig. S1). Genes that express the proteins identified in this study (MUC19 and LIPF) were selected and the parameters chosen following the protocol given by the website.

### Proteomic analysis

Guanidine hydrochloride (GuHCl) 8 M was added to the saliva samples to a final GuHCl concentration of 4 M. Samples were treated with 10 mM DTT in 50 mM Hepes, pH 8.0, at 65 °C for one hour to denature proteins and reduce disulfide bonds, followed by alkylation with 50 mM iodoacetamide in 50 mM Hepes in the dark at room temperature for one hour. The samples were submitted to digestion with sequencing grade modified trypsin (Sigma) at a 1:50 ratio of trypsin:protein sample for sixteen hours at 37 °C. Digestion was halted using 5ul of 10% trifluoroacetic acid (TFA). Digested samples were desalted using C18 ziptips (Pierce, Thermo Fisher Scientific) following manufacturer's protocol and resuspended in 0.1% formic acid. Samples were analyzed in an EASY II-nanoLC system (Thermo Scientific, Bremen, Germany) coupled to an LTQ-Orbitrap Velos mass spectrometer (Thermo Scientific). The peptides were loaded onto an in house packed C18 (Jupiter 10 μm beads, Phenomenex Inc, Torrance, CA) pre-column (100 μm ID x 360 μm OD) and separated on an in house packed C18 (ACQUA, 3 μm beads, Phenomenex Inc, Torrance, CA) analytical column (75 μm ID x 360 μm OD) on which were separated over a 120 min gradient using solvent A (0.1% formic acid in water) and solvent B (0.1% formic acid in acetonitrile). The gradient consisted in a constant flow of 200nL/min with an initial gradient of 5% to 30% B from 0 to 85 min, 30% to 90% B from 85 to 95 min, 90% B from 95 to 105 min, 90% to 5% B from 105 to 107 min, 5% B until 120 min. The mass spectrometer was operated in full scan mode where the top 10 most intense precursor ions were selected in a data-dependent acquisition mode and nanospray voltage at 2.3 kV. The MS1 were acquired in FTMS from 300 to 2000 m/z at a resolution of 30.000, and the spectra of the product ions with the MS2 resolution of 7.500. The MS2 was performed in ITMS with CID method at a normalized collision energy of 35.0, isolation width of 2.0 m/x, default charge state of 2, activation Q of 0.250, and activation time of 10.000, and charge states equal to 1 and unassigned states were rejected.

### Protein identification and quantification

Mass spectrometer Raw files were processed using MaxQuant software (version 1.6.1.0) against the Mus musculus database downloaded from Uniprot. The quantification was performed using the Label-free quantification (LFQ) algorithm from MaxQuant from which the normalized intensities were used. The software was set as the first search peptide mass tolerance in 20 ppm, the main search peptide mass tolerance in 4.5 ppm. The digestion enzyme was set as trypsin, cysteine carbamidomethylation as fixed modification, while methionine oxidation and N-terminal acetylation was set as variable modifications. The data output from MaxQuant was analyzed using Perseus software (version 1.5.8.5).

### Bioinformatic analysis

All acquired proteome data was analyzed in order to classify the functional enrichment of protein profiles based on biological processes, molecular function, cellular components, and cellular pathway using the online platforms GO (Gene Ontology, geneontology,org) and PantherDB (Protein Analysis Through Evolutionary Relationships) classification system^47^.

### Statistics analysis

For statistical quantitative analysis, the “protein groups” files from MaxQuant were input into the Perseus software, where the LFQ intensity data were processed through the filtering out contaminants, reverse sequences (decoys) and “only identified by site” proteins… To identify the interactors a two samples t-test was performed for each comparison. The parameter used for the test was ‘Permutation-based FDR’, with FDR being 0.05 and S0 = 2. To visualize the t-test significant proteins a volcano plot was obtained.

## Supplementary Information

Supplementary Information.

## Data Availability

The datasets analyzed during the current study are available in the ProteomeXchange Consortium via the PRIDE^48^ partner repository with the dataset identifier PXD031219.
