# Supplementary material for: Identification of early biomarkers in saliva in genetically engineered mouse model C(3)1-TAg of breast cancer
Source: Sci Rep. 2022 Jul 7;12:11544. doi: 10.1038/s41598-022-14514-1 (PMC9263110; doi:10.1038/s41598-022-14514-1)
Supplement: Supplementary file 1 — Supplementary Information. [file 41598_2022_14514_MOESM1_ESM.docx]

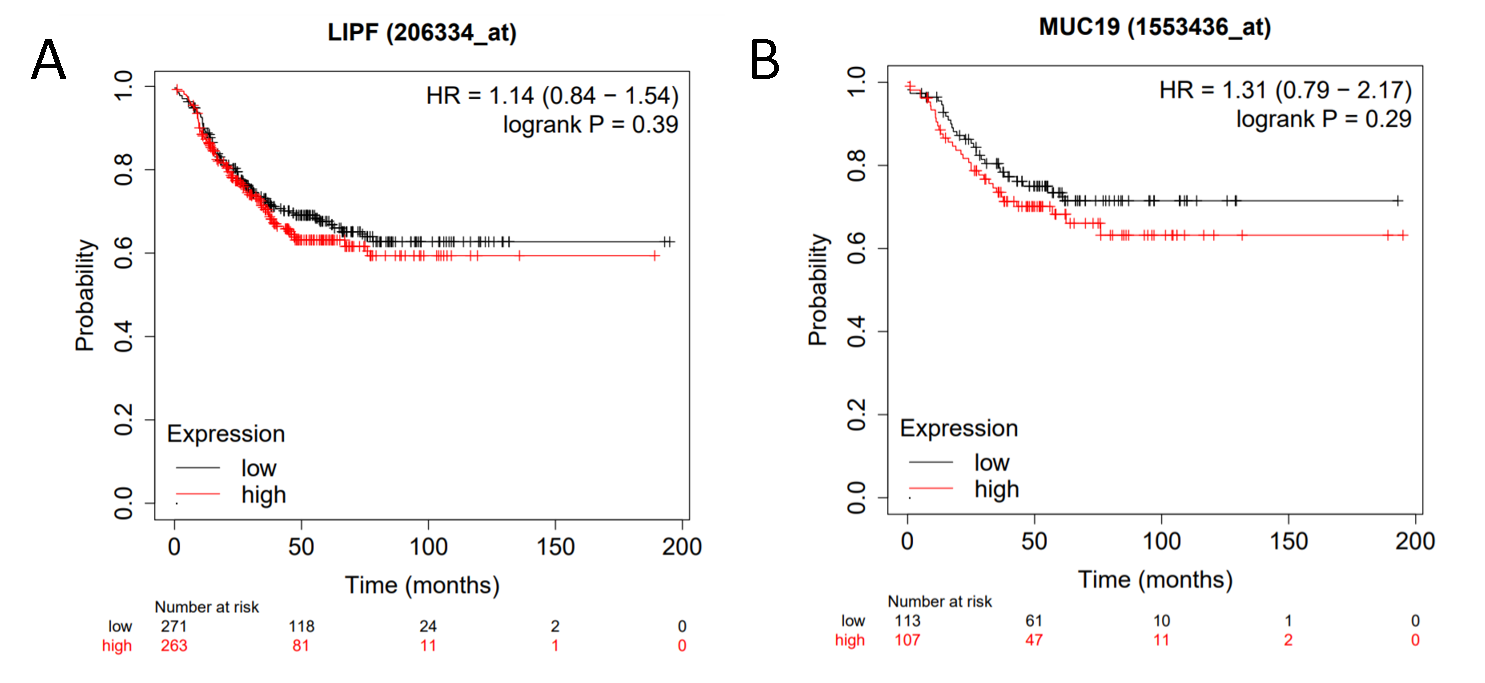


**Supplementary Figure S1.** High expression of LIPF and MUC19 correlates with worse prognosis in tumor breast. The survival curve of LIPF and MUC19 in triple-negative breast cancer patients show that (**a**) high LIPF expression and (**b**) MUC19 is correlated to a lower survival rate.
